# Process evaluation of two large randomized controlled trials to understand factors influencing family physicians’ use of antibiotic audit and feedback reports

**DOI:** 10.1186/s13012-024-01393-5

**Published:** 2024-09-16

**Authors:** Jennifer Shuldiner, Meagan Lacroix, Marianne Saragosa, Catherine Reis, Kevin L. Schwartz, Sharon Gushue, Valerie Leung, Jeremy Grimshaw, Michael Silverman, Kednapa Thavorn, Jerome A. Leis, Michael Kidd, Nick Daneman, Mina Tradous, Bradley Langford, Andrew M. Morris, Jonathan Lam, Gary Garber, Jamie Brehaut, Monica Taljaard, Michelle Greiver, Noah Michael Ivers

**Affiliations:** 1 Women’s College Hospital Institute of Virtual Care and Systems Solutions, Women’s College Hospital, Toronto, ON Canada; 2 Institute for Health Policy, Management and Evaluation, University of Toronto, Toronto, ON Canada; 3 Lunenfeld-Tanenbaum Research Institute, Sinai Health Catherine Reis, Women’s College Hospital, Toronto, ON Canada; 4 Women’s College Research Institute, Women’s College Hospital, Toronto, ON Canada; 5 ICES, Toronto, ON Canada; 6 Unity Health Toronto, Toronto, ON Canada; 7 Public Health Ontario, Toronto, ON Canada; 8 Dalla Lana School of Public Health, University of Toronto, Toronto, ON Canada; 9 Ontario Health, Toronto, ON Canada; 10 Michael Garron Hospital, Toronto, ON Canada; 11 Toronto East Health Network, Toronto, ON Canada; 12 Ottawa Hospital Research Institute, Ottawa, ON Canada; 13 Department of Medicine and Infectious Diseases, Division of Infectious Diseases, Western University, London, ON Canada; 14 Sunnybrook Health Sciences Centre, Toronto, ON Canada; 15 Nuffield Department of Primary Care Health Sciences, The University of Oxford, Oxford, UK; 16 Centre for Future Health Systems, The University of New South Wales, Sydney, Australia; 17 Department of Family and Community Medicine, University of Toronto, Toronto, Canada; 18 University of Toronto, Toronto, ON Canada; 19 Sunnybrook Research Institute, Toronto, ON Canada; 20 Leslie Dan Faculty of Pharmacy - University of Toronto, Toronto, ON Canada; 21 Mount Sinai Hospital, Toronto, ON Canada; 22 Ontario Health Ontario, Toronto, Canada; 23 Department of Medicine and Department of Epidemiology and Public Health, University of Ottawa, Ottawa, Canada; 24 Department of Medicine, University of Toronto, Ottawa Hospital Research Institute, Ottawa, Canada; 25 Department of Family and Community Medicine, Women’s College Hospital, Toronto, ON Canada; 26 Institute for Health Systems Solutions and Virtual Care, Women’s College Hospital, Toronto, ON Canada

**Keywords:** Audit and feedback, Antibiotics, Process evaluation

## Abstract

**Background:**

Unnecessary antibiotic prescriptions in primary care are common and contribute to antimicrobial resistance in the population. Audit and feedback (A&F) on antibiotic prescribing to primary care can improve the appropriateness of antibiotic prescribing, but the optimal approach is uncertain. We performed two pragmatic randomized controlled trials of different approaches to audit and feedback. The trial results showed that A&F was associated with significantly reducing antibiotic prescribing. Still, the effect size was small, and the modifications to the A&F interventions tested in the trials were not associated with any change. Herein, we report a theory-informed qualitative process evaluation to explore potential mechanisms underlying the observed effects.

**Methods:**

Ontario family physicians in the intervention arms of both trials who were sent A&F letters were invited for one-on-one interviews. Purposive sampling was used to seek variation across interested participants in personal and practice characteristics. Qualitative analysis utilized inductive and deductive techniques informed by the Clinical Performance Feedback Intervention Theory.

**Results:**

Modifications to the intervention design tested in the trial did not alter prescribing patterns beyond the changes made in response to the A&F overall for various reasons. Change in antibiotic prescribing in response to A&F depended on whether it led to the formation of specific intentions and whether those intentions translated to particular behaviours. Those without intentions to change tended to feel that their unique clinical context was not represented in the A&F. Those with intentions but without specific actions taken tended to express a lack of self-efficacy for avoiding a prescription in contexts with time constraints and/or without an ongoing patient relationship. Many participants noted that compared to overall prescribing, A&F on antibiotic prescription duration was perceived as new information and easily actionable.

**Conclusion:**

Our findings indicate that contextual factors, including the types of patients and the setting where they are seen, affect how clinicians react to audit and feedback. These results suggest a need to test tailored feedback reports that reflect the context of how, where, and why physicians prescribe antibiotics so that they might be perceived as more personal and more actionable.

**Trial registration:**

Clinical Trial registration IDs: NCT04594200, NCT05044052.

**Supplementary Information:**

The online version contains supplementary material available at 10.1186/s13012-024-01393-5.

Contributions to the literature

- This study leverages a pragmatic trial design and a theoretical informed process evaluation to enhance our understanding of why and how family physicians use antibiotic audit and feedback.
- This large-scale process evaluation evaluated three design modifications of antibiotic audit and feedback.
- The study identified unique factors creating information-intention gaps (when A&F fails to encourage recipients that change is necessary), and intention-behaviour gaps (when intentions formulated in response to A&F are not rendered into action).

## Background

Primary care physicians prescribe most antibiotics in humans, making this prescriber group crucial partners in antimicrobial stewardship efforts [1–3]. Audit and feedback (A&F) can act as an effective intervention to reduce unnecessary antibiotic use in primary care [4–7]. Numerous trials show that feedback that shows how health professionals’ prescribing practices compare to those of their peers can be an effective intervention for reducing antibiotic prescribing rates among family physicians [4, 6, 8]. However, research is needed to examine ways to optimize the effects of A&F [9].

We conducted two interrelated, province-wide trials of A&F in Ontario, Canada, with embedded process evaluations [10], to reduce antibiotic prescribing by family physicians. Those trials involved tests of different ways of designing the A&F interventions, which we described in detail in a prior manuscript. Briefly, we tested three variations in intervention design across the two trials: (i) raw versus adjusted data in the A&F to help recipients see how their antibiotic prescribing compares to other physicians with similar patients; (ii) information about the futility of antibiotics for conditions that are primarily viral versus emphasis on the potential harms of antibiotics; and (iii) provision of mailed ‘viral prescription pads’ [10] as a communication tool to help recipients act upon intentions of avoiding antibiotics.

The A&F reduced antibiotic prescribing by 5% over six months compared to no intervention. The mean (standard deviation) antibiotic prescribing rate was 59.4 (42.0) in the control arm and 56.0 (39.2) in the intervention arm (adjusted relative rate (RR) 0.95 (95%CI,0.94 to 0.96). However, no differences were found across the various intervention arms. Results were consistent at 12-months post intervention.

The full trial results will be reported elsewhere; here, we report on the qualitative process evaluation in which we sought to understand the outcomes observed and what can be done to optimize A&F for antibiotic prescribing.

## Methods

### Study design

We used qualitative methods in this embedded process evaluation to understand how and why the intervention worked (or did not work) as intended. We combined participants from both trials since both targeted antibiotic prescribing amongst family physicians and were delivered simultaneously and in contexts. This study received research ethics approval from the Women’s College Hospital Research Ethics Board. The reporting of this qualitative process evaluation adheres to the COnsolidated criteria for REporting Qualitative research (COREQ) reporting standards (Appendix 1).

### Theoretical framework

The Clinical Performance Feedback Intervention Theory (CP-FIT) offers the most comprehensive theory on the conditions for optimal A&F [11]. It is a product of a qualitative synthesis of 65 studies that culminated in a healthcare-specific theory of A&F. The theory posits that the effects of A&F can be summarized by three propositions: (1) health care professionals and organisations have a limited capacity to engage with feedback, (2) these parties have strong beliefs about how patient care should be provided that influence their interactions with feedback, and (3) feedback that directly supports clinical behaviours is most effective. CP-FIT guided our thinking regarding the mechanism of action of A&F in clinical practice and factors that influence its effects. It was used to inform the development of the interview guide and the analysis [12].

CP-FIT states that effective feedback works in a cycle of sequential processes. We explored this process of feedback interaction, then recipient perception and acceptance of the feedback, followed by intention, and then behaviour change for clinical performance improvement. The theory stipulates that progress through the cycle will be weakened or halted entirely if any individual stage fails. CP-FIT highlights three types of variables that operate through common explanatory mechanisms to influence whether and how health professionals respond to A&F: the feedback intervention itself, characteristics of the feedback recipient, and contextual factors affecting the clinical environment.

### Context and setting

Ontario has a population of over 15 million people where the majority of primary care is delivered by family physicians. A universal government-funded insurance plan without deductible or co-pay covers visits to family physicians. Medications, including antibiotic prescriptions, are covered for those on social assistance, those under 25 with no private (employer-funded) insurance, and all those above age 65.

The trials (Table 1) were conducted with *Ontario Health* and *Public Health Ontario.* Ontario Health— an agency created by the Government of Ontario with a mandate to connect and coordinate the province’s health care system to help ensure that Ontarians receive the best possible care—provides A&F to physicians who voluntarily sign up for their “*MyPractice*: Primary Care” reports. Approximately 4750 (of 9,500 eligible) Ontario family physicians signed up to receive these reports during this study. These are multi-topic reports with aggregated (physician-level) data, sent twice yearly via email using data collated from the Institute for Clinical Evaluative Sciences (ICES, a custodian to a data repository with patient and physician-level, coded and linkable health data sets in Ontario, Canada). ICES data includes publicly funded administrative health services records for the Ontario population eligible for universal health coverage (≈ 98.5%). However, dispensing data are complete only for patients 65 and older.

**Table 1:** Characteristics of Public Health Ontario Trial and the Ontario Health Trial

	Public Health Ontario Trial	Ontario Health Trial
Trial Design	4:1 to intervention or control	Cluster-randomized by practice, 1:1 to different intervention arms
Trial Arms	Physicians in the intervention arm of this trial received one of four versions of a personalized antibiotic A&F: feedback featuring case-mix adjusted versus unadjusted comparator and/or emphasis or not on harms of antibiotics.	A&F alone or a stack of “Viral Prescription Pad” mailed to their office as well as added emphasis in their report on use of the pad.
Audit and Feedback type	Single topic audit and feedback	Multi-topic audit and feedback
Intervention delivery	Letter sent via post to clinic address	Email with link to audit and feedback, with or without mailed stack of “viral prescription pads”
Opt-in for intervention	No sign up required	Sign-up required
Audit and feedback data on initiation	Prescription rate per visit and a graph with prescribing rates and their comparators (25th and 50th percentiles).	Prescription rate per visit and a graph with prescribing rates and their comparators (50th percentiles)
Audit and feedback data on duration	Percentage of prescription over 7 days	Percentage of prescription over 7 days and a graph with prescribing rates and their comparators (50th percentiles)
Audit and feedback data initiation – “High prescribers”	25th percentile	50th percentile

Public Health Ontario (PHO)—an agency of the provincial government responsible for providing scientific and technical advice on matters of public health concern— sent A&F specifically about antibiotic prescribing to family physicians who did not sign up for the *MyPractice* report from Ontario Health. The PHO A&F reports also used data held at ICES to link prescriber characteristics, including patient volume, and patient characteristics, including comorbidities, to antibiotic prescription data [10].

### Recruitment

All A&F recipients were given a process evaluation survey that included an invitation to participate in an interview. Participants were asked to write their contact information so the study team could follow up. From the physicians who indicated interest in participating in an interview, participants were purposely sampled from defined strata to allow maximum variation across age, gender, experience (i.e., years worked as a family physician), and clinical context subgroups (i.e., walk-in physician, family health team, emergency). We also purposively sampled from each of the following groups: (i) PHO Trial, adjusted comparator, (ii) PHO Trial, unadjusted comparator, (iii) PHO Trial, harms emphasis, (iv) PHO Trial, no harms emphasis, and (v) OH Trial, mailed viral prescription pad/emphasis.’

An information letter introducing the research team and outlining the purpose of the study and an informed consent form were provided via email. All physicians who completed the interview were provided an honorarium of $100 as an electronic gift card, recognizing the time required to complete the interview. Participants were screened to confirm they had read the A&F letter before the interview over email or at the beginning of the interview if not previously answered. Recruitment ceased when data saturation was achieved, defined as the point in data collection and analysis when new incoming data produced little or no new information to address the research questions.

### Data collection

Brief demographic questions were asked at the beginning of the interview, including the type of A&F received, gender, years in practice, type of practice (Interprofessional practice, Community Practice, Walk-in clinic, Other), location of practice (urban, rural), and the average number of patients seen per day. Interviews were conducted between 1 February 2022 and 5 April 2022 by two non-clinician researchers (ML and JS) trained in qualitative methods. The interview guide explored CP-FIT theory constructs (Appendix 2) and the different aspects of the A&F letter (e.g., comparators, duration data, and harms information). All interviews were conducted on Zoom (Zoom Video Communications, San Jose, CA), recorded and transcribed verbatim and entered into NVivo (QSR International), a qualitative analysis software program. Only the researchers conducting the interview were present and the interviews were scheduled for 60 min.

### Analysis

We used reflexive thematic analysis that involved a constant comparative method, with our research questions guiding our analysis of transcripts [13]. In this process, we applied inductive open coding, involving a preliminary reading of full transcripts and generating initial descriptive codes- paraphrasing the text using participants’ own words. Transcripts were coded by four team members (JS, ML, MS, CR). All four team members coded the first four transcripts independently using open codes. The coders met to iteratively develop a mutually agreed upon analytical framework, which was applied to all transcripts using focused coding in NVivo. Our analysis considered physicians from both trials together.

We were interested in both high and low prescribers for data looking at how recipients responded to intervention factors (i.e., viral prescription pad, adjusted comparator). However, when gathering insights to inform future interventions, the response of A&F recipients without substantial room for improvement isless important from a public health perspective. Therefore, majority of our analysis focused on physicians who were described as “high prescribers” in their A&F report. “High prescribers” prescribed more than the target expressed in their A&F report (above the 25% target in the PHO trial and above 50% in the OH trial.

The team mapped codes onto the CP-FIT theory constructs, and broader themes were created by grouping codes based on the CP-FIT constructs. Subthemes and their relationships were reviewed, mapped, and discussed with the larger team. Finally, we examined the data in the context of the CP-FIT explanatory mechanisms that influence the different stages of the CP-FIT cycle. Specifically, we considered factors that affected progress through the CP-FIT stages for two different behavioural targets of the A&F reports: antibiotic prescription initiation and antibiotic prescription duration. Comparing factors influencing these behaviours could illuminate why some metrics seem more amenable to improvement via A&F than others. We also compared data, codes, and themes across important characteristics (e.g., type of clinic, rural vs. urban, age of physician).

## Results

We conducted a total of 45 interviews. Of these, 12 of 39 who expressed interest were participants in the Ontario Health trial, and 33 of 146 were in the Public Health Ontario trial (Table 2). Twenty-six participants (58%) self-reported as male and 28 (62%) worked in urban locations. The average years of experience was found to be approximately 20 years.

**Table 2:** Characteristics of physicians who participated in an interview

Physician Characteristics	PHO Trial, *N* = 33 *N* (%)	OH Trial, *N* = 12 *N* (%)
Male	19 (58)	7 (58)
Years in Practice, median (SD)	12 ± 11.37	21 ± 15.60
Practice Location		
Urban	22 (67)	6 (50)
Semi-Urban	7 (21)	3 (25)
Rural	4 (12)	3 (25)
Practice Setting^a^		
Family practice or outpatient clinic	19 (58)	3 (25)
Family Health Team	6 (18)	6 (50)
Family Health Organization	1 (3)	2 (17)
Walk-in clinic	7 (21)	1 (8)
Emergency or urgent care	10 (30)	0 (0)
Long-term care	4 (12)	1(8)
Hospital-based Practice	6 (18)	1(8)
Other	4 (12)	0 (0)
Roster size, median (SD)^b^	1035 ± 410.88	1400 ± 320.64
Patients seen per day, median (SD)	25 ± 10.24	21 ± 6.60
“High prescribing”	23 (70)	6 (50)

^a^11 physicians in the PHO trial and 2 physicians in the OH trial worked in multiple settings

^b^Numbers are based on physicians’ estimates and may not be exact. Seven physicians in the PHO trial said their Practice does not have a roster. Thirteen physicians in the PHO trial and 4 physicians in the OH said they were unsure of how large their roster was

### Exploring lack of effect differences across trial arms: reactions to intervention components

The following sections explore physician reactions to the intervention components. Some of these items were explicitly tested in the trials, and others were not, but participants’ responses to all intervention components helped explain the observed effects.

#### Comparator

Many of the physicians interviewed received an A&F letter with comparator data adjusted for patient and practice characteristics. However, many still described how the comparison provided in the letter did not apply to their unique practice (benchmarking). Physicians emphasized how variation in access to urgent appointments, practice settings (urgent care, emergency, long-term homes), or practice in rural areas could contribute to more antibiotic prescribing. They also discussed how the characteristics of their patient population might necessitate different prescribing patterns (e.g., older patients, younger patients, or patients with more acute presentations).

“I think if we can compare my own particular work situation with another family physician, urgent care, walk-in clinic physician, apple for apple, I think that would be more valid and more reliable data.” – PHO 956 adjusted comparator, harms information, high prescriber.

“I see people the same day. Most of my peers don’t. When we see people the same day, we see more acute presentations of illness. Whereas my colleagues aren’t often seeing people in acute infectious periods and will have less opportunity to provide antibiotics, and those people are more likely to seek antibiotics elsewhere.”- PHO, 4255, adjusted comparator, harms information, high prescriber.

“The expectation of using antibiotics according to guidelines is based on studies that were done in a controlled manner – but it’s not always possible in real life situations when there is uncertainty and when there’s risks that come with not prescribing. Sometimes it’s fine to take the risk, because you have a backup plan, or you can educate the patient what to do if it develops a certain way. But sometimes, you can’t because the patient is too frail, or there’s too many complexities and you don’t want to take chances. So, you basically treat them with everything to keep them from decompensating or ending up in the Emergency.” – PHO Trial, 4299, adjusted comparator, harms information, high prescriber.

#### Harm information

The physicians did not emphasize antibiotic harm data’s role in how they responded to the A&F. In general, we heard from physicians that the harms information was not new to them but that having the specific data was helpful when having antibiotic prescription conversations:

“I thought it was great to have numbers because I know these things. But it’s helpful to be able to quote a number to a patient. So that was great.” – PHO 3381 unadjusted comparator, harms information, high prescriber.

“The information around patient harms was new in its level of detail and breadth. That’s the thing - it’s not just all resistance. It’s about people’s terrible diarrhea and their C. difficile and all that stuff. For all that, this was useful.” – PHO 3519, adjusted comparator, harms information, high prescriber.

“I do discuss the resistance of bacteria to antibiotic, I’ve spoken to patients about this. I tell them, when it comes to a sinus infection, there’s a lot of information that using a nasal spray is better than antibiotic because it works topically, drains your nose. And again, the harm of the antibiotic, that could cause too a next generation that we may not have a good antibiotic to treat our children in future.” – PHO 604 adjusted comparator, no harms information, high prescriber.

#### Achievable target

Physicians’ acceptance of the data indicating an opportunity for improvement hinged on the recipient’s perspective of the comparator used in the A&F, including the ‘achievable target.’ Many physicians in the PHO trial (where the achievable target was set at the lowest 25% of prescribers) did not accept this because they believed it was either unachievable or undesirable. Others described being satisfied that they were close to the average prescriber. The findings related to this are elaborated in Table 3.

**Table 3:** Belief statements that relate to the comparator featured in the audit and feedback letter

Belief statement	Quote
The target is not meaningful *(benchmarking)*	“People like me are never going to believe we’re going to get down to the one quarter quartile. Id be thrilled if I even got down to the 50% quartile” – PHO Trial, 537 “I don’t disagree with the target. But I also can’t see 50% of my prescriptions being totally useless. Because if I did, then what am I doing here? But I do think that I could reduce - there are areas that I - when I’m looking closely at it, that I think I can do better. And prescribe less. And really encourage people to wait.” - PHO 4255 “I’m more than willing to try and do better compared to my peers. I’m certainly not going to put a ton of effort trying to get into what somebody who crunched numbers says is an achievable target, because they could have picked anything…I have no idea what they’re using as a goal and whether it’s even semi-achievable for me.” – PHO Trial, 537
Being near-average is desirable *(Performance level)*	“I was happy to know that at this point, I am below the average prescriber, I’ll be at somewhat above the achievable target. And I felt good about it.” – PHO Trial, 3470 “I’m below the average. I’m OK with that. So I didn’t concentrate as much on the achievable target. Is the achievable target the ideal amount you should be prescribing? Or is it just, let’s try to aim for this?” – PHO Trial, 4257 “Well, I’m doing better than the average.” If you concern the number of prescriptions as – if the average is fairly good then I’m doing a little bit better than that, so that was my thought. – PHO Trial, 1797
Some physicians are under-prescribing *(Clinical appropriateness)*	“The lowest prescribing quartile, physicians, maybe they’re under prescribing, who knows. But overall, I do believe that there is an issue with over prescribing. So at least to reach towards that target is a good idea.” – PHO Trial, 4772

“*But it’s just an arbitrary number that you guys decided that if you can reach what 25% of other people do*,* that that should be a goal that you should set for? How do you know that 25% of people aren’t prescribing enough*.” – PHO Trial, 4675 adjusted comparator, no harms information, high prescriber.

#### Responses to antibiotic duration data

Physicians reacted differently to data about the proportion of their antibiotic prescriptions for a prolonged duration than to data about antibiotic prescription rate. They were more *accepting* and described learning new information regarding evidenced-based practice for duration.

“*I was higher by almost 30%. So that was quite an eye opener*,* because I guess I tend to use a lot of antibiotics*,* that is*,* for 10 days*,* rather than the seven. So that was a learning point for me*,* to use it more judiciously in that sense*,* shorter courses may be just as effective*.”– OH Trial, 4, low prescriber.

“*It was awesome. It was*,* some of it felt brand new to me*,* which is kind of embarrassing. But I guess we’re always learning. I was really excited. I was like*,* fantastic. Somebody’s done the digging for me*,* summarized the data*,* and here it is. Let’s go.*” – PHO Trial, 3381, unadjusted comparator, harms information, high prescriber.

Many physicians described their *intentions* and changing *behaviours* to incorporate new duration recommendations after they received feedback. The A&F data was viewed as compatible with their views on patient care and immediately actionable. Some explained that this was easy because no pushback was expected from patients; others said that they would make the change tentatively and monitor outcomes, emphasizing once again the need for a case-by-case consideration.

“I dropped the duration of my antibiotic prescriptions. Basically, as soon as I read that chart and looked at some of the references that they’re referring to”. – PHO Trial, 3881 unadjusted comparator, harms information, high prescriber.

“That’s pretty easy. That’s never – almost never a fight, its just keeping up to date with the latest recommendations, so this is helpful to update us and making sure our resources are up to date, and that’s pretty easy. Very rarely do people care about how long. They usually just want the antibiotic for any duration” – PHO Trial, 1088 unadjusted comparator, no harms information, high prescriber.

“I thought I was doing durations appropriately based on each presentation. This study has given me an opportunity to reflect…I am re-evaluating. So I can see how the changes actually work in real life settings.- PHO Trial, 1366, unadjusted comparator, no harms information, high prescriber.

“I know that data say you could do three days [for UTIs], but clinically, I have seen too many recurrences with that shorter course. And I make the judgement call to give my patients five days. I adjusted my Practice, I didn’t like the outcome, and then I went back. – OH Trial, 26, low prescriber.

#### Viral prescription pad

A version of the viral prescription pad was provided to all recipients of the feedback letter. In the PHO trial, information regarding the pad was included in the feedback letter, and in the OH trial, we tested differences between those who received a pad in the mail versus those who did not. For OH participants, we only interviewed physicians who received the mailed viral prescription pad. In general, we found that physicians described how they liked having instructions to give to the patient “it’s a good tool” (OH 163) and thatpatients like to receive something formal. Overall, they found it useful when a patient was asking for antibiotics and they used it to help guide conversations regarding antibiotics.

However, physicians also described limited engagement with the pad (i.e., using it)and that there were limited opportunities or examples where the viral prescription pad was handed to the patient.

“Although I haven’t used that viral prescription handout, it was nice to reference it on the phone with patients and just like, say, for bronchitis, the duration of a cough is ten to twenty-one days, right, and to kind of feed that back to patients and avoid any antibiotics.” – OH 126, high prescriber.

Even times when physicians described that the viral prescription pad was integrated within the EMR it was still not accessed. Physicians described that it did not fit within their existing routine, especially their virtual workflows:

“So we did end up looking at it, and we actually put it into our EMR system. To be honest, I haven’t used it. I guess it’s difficult too, because again, most of these scenarios, if I am seeing them and following up with them, by phone, or virtually afterwards. “ – PHO 4756.

### Optimizing future antibiotic A&F interventions

**Fig. 1:**
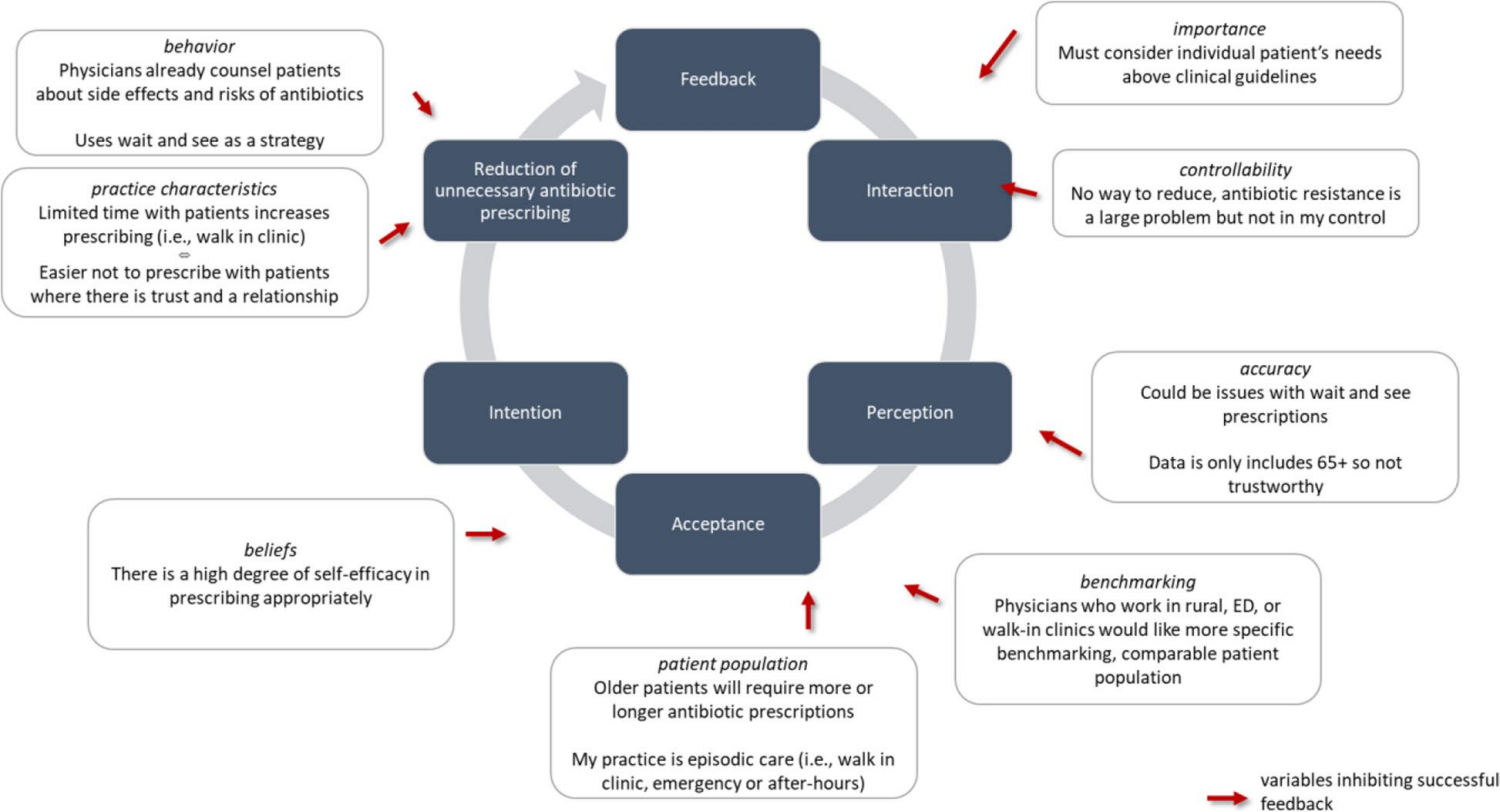
Adapted Clinical Performance Feedback Intervention Theory feedback loop: determinants of antibiotic prescribing

Figure 1 summarizes the factors influencing progress through the stages of the CP-FIT feedback loop, which describes how recipients interact with A&F, why they might use it to form intentions to change, and whether they might act upon those intentions. We elaborate our findings below in two sections that explain why feedback might fail to encourage recipients that a change in antibiotic use is necessary (creating an “information-intention gap”) and why intentions might not be translated into action (an “intention-behaviour gap”).

Factors that affect engagement with antibiotic A&F and formation of intentions to change were distinct from those that affect the use of the A&F to change antibiotic prescribing. Overall, we found that “High prescribing” physicians described various factors that inhibited them from completing the feedback cycle (i.e., describing intention or perceived behavior change in antibiotic prescribing). In general, those who prescribed fewer antibiotics were more accepting of the feedback and were reassured by their feedback results. Table 4 organizes key findings that influence progression through the feedback loop and supporting quotes into variables related to (i) the feedback design, (ii) the recipients, and (iii) the context, keeping with the categories of variables used in CP-FIT.

**Table 4:** Themes found relating to CP-FIT constructs found to influence whether and how family physicians responded to A&F intervention

	Belief Statements	Quote
**Feedback variables**	No way to reduce, antibiotic resistance is a large problem but not in my control (*controllability*)	“I’m already doing everything I can to prevent antibiotics from being used inappropriately” - PHO trial, 3483 “I felt I was judicious about my antibiotics, and I always try to persuade people out of it. So I don’t know if it’s going to make a difference on how I interact with patients because I felt I was already doing my best at that” – OH Trial, 28, high prescriber “Well, I mean, obviously it’s [resistance] a problem that has to be addressed. It’s a long time coming. I mean, it’s - there’s so much damage that has been done, but what are our choices? We have to preserve what we have right now. And a lot of it, well, it stems from our prescribing habits. And I mean, over the counter use in other countries, some of these antibiotics, they can just buy off the shelf, that’s - well, hopefully that doesn’t happen here, but we should do what we can here to sort of avoid that” – OH Trial, 4, low prescriber
	Must consider individual patient above guidelines (*importance*)	“You can recommend anything but for the specific patient it’s what –, how they’re doing is what counts and not what statistics tell us to do” – PHO Trial,1797 “You try to educate them the best you can but then there are times where – sometimes we just take them to the hospital, then I just end up giving it [antibiotics] because it doesn’t make sense for them having to go to the hospital, for example. So yeah, there are exceptions but usually I would say if the patient is reasonable enough, and I’m able to speak to them, then I usually do prescribe based on the guidelines” – OH Trial 25, low prescriber
	Trustworthiness of the data as limited to patients 65 years of age and older (*accuracy*)	“My thoughts on the data… still questionable, because I am reading here that antibiotic prescriptions in patients over – 65 and over is highly correlated with overall antibiotic use for all age groups among Ontario family physicians. I see that even like in bold, italicized letters. I’m not sure I particularly can agree with that claim, to be honest with you” – PHO Trial 956,
	Unclear impact of delayed prescribing (*accuracy*)	“I mean, I guess the intention in which we prescribe is different than what prescriptions are filled at the pharmacy. So again, a lot with my elderly patients, they might fill it, leave it at home in their cupboard, or take it with them on vacation, if they have recurrent UTI’s, or something like that. And they want a prescription for that. That does happen more frequently, again, in that patient population. So I don’t feel like it’s necessarily as reflected as to what people are actually taking.” - PHO 4756 “Sometimes I follow up with patients after an encounter where I’ve provided a delayed prescription, I ask them did you end up needing it and fortunately they say no, but they did fill it. I’m not saying that happens all the time, but it happens a number of times for sure.” - OH Trial 163, high prescriber
	Physicians who work in rural, ED, or walk-in clinics would like more comparable patient population *(benchmarking)*	“I think it would also be good to compare different regions that have higher rates of different infections than others. And so maybe it’d be more beneficial based on your location specifically and not necessarily all of Ontario.” – OH Trial 26, low prescriber “I think the region might affect it a little bit, but not to the extent that emerge versus an office would. So I think any physician who works purely emergency medicine would be a good comparator in terms of rates of prescribing.” – PHO, 3381 “So it struck me, that I didn’t see any regionalisation. When we’re given our stats for preventative medicine, they usually regionalise it for us. You know, how are you compared to other Northern Ontario doctors, because things are different up here.” -PHO, 537
**Recipient variables**	High degree of self-efficacy in prescribing appropriately, but also acknowledge difficulty under pressure from patient and limited time in patient encounter *(beliefs)*	“I do think it [time constraints] impacts to some extent. It shouldn’t and I try to minimize it and I don’t let it happen often, but it may occasionally lead to unnecessary antibiotic prescribing. I think it would be rare instances, but time pressures do impact these things.” - OH Trial 163, high prescriber “So I always am conscious about trying to prevent antibiotic resistance so it’s something that I always have in the back of my mind, but sometimes you know we may be pressed for time, it may be easier just to write a script than you know have the patient be sent home. It may be the easier way out but I try to make sure that I do educate patients, that if there is no indication to prescribe an antibiotic I’d rather take the extra few minutes to educate a patient, not just give the patient a script, rather than just take the easy out.” - PHO 1027
	Common behaviors include delayed prescriptions *(behavior)*	“One way of getting around it is to say OK, well here’s a prescription, but don’t fill it. You know if this happens by that day then fill it. In the hopes that I’ve bought some time for them, and maybe they’ll get better. But if they’re worse they don’t have to go through the ordeal of getting a hold of me to go through the whole assessment again.” - PHO Trial, 3283 “What I’ll do sometimes is I’ll give them a prescription, I’ll say, I’ll give you the prescription, but you must wait three days and see. If it gets better, you don’t need to fill it in. So then the ball is in their court. They have a prescription; they won’t have to show up to emerge if it’s the weekend. So, if it does get any worse, and I give them the criteria, then they go and fill it. But I tell them you don’t need to fill it now. Be safe, be sort of reassured that if you do need it, you have it.” – OH Trial 65, high prescriber
	Physicians already counsel patients about side effects and risks of antibiotics (*behavior*)	“I usually explain to the patient that I believe it’s a viral illness and antibiotics aren’t effective against viruses. And that the potential for side effects and harm with antibiotics was greater in my opinion than the potential for it to be helpful. I often say, if I was in your situation, I wouldn’t take the antibiotics. I would wait and see.” – PHO Trial, 2924 “I acknowledge their frustration with the symptoms. So that would be one. Number two, my hesitancy about prescribing antibiotics is not to make their lives difficult or anything, but just that it’s not going to speed up their recovery. Number three, harms, from diarrhea to anaphylaxis, to antibiotic resistance. And then four, I describe the symptoms that they should watch for.” – OH Trial 126, high prescriber
**Contextual variables**	Limited time with patients increases prescribing (i.e., walk in clinic) *(Practice characteristics)*	“I think it’s a lot faster to just give them what they want in a time crunch, so that’s a definitely competing factor in the emergency, but when those waits are long you have less and less and less fight in you and that’s just a reality.”- PHO Trial, 1088 “This is the area where I still struggle with, especially in a walk-in clinic setting where there are too many patients to see and limited time. This is when I’m on the fence. Usually if it’s clear that there is no infection then I would not prescribe it, but if I’m on the fence then I would err towards the side of prescribing something, whether it is to start something or a delayed prescription. So time constraints are certainly a major barrier I find to be able to prescribe antibiotics appropriately.” – OH Trial, 25, low prescriber “For the most part, yes. Yes, that’s what I do [counsel patients on risks of antibiotics], although let’s not forget in a very busy, urgent care, walk-in clinic, there might not be enough time to go through lengthy explanations. But for the most part that’s what I strive to do and to let them know if it’s not better within so and so timeframe, come back, and we can always reassess your situation” - PHO Trial, 956
	My Practice is episodic care *(Patient population)*	“ I do purely walk-in clinic. The type of patient I see, it’s not the type of patient that a regular family practice they see in their office….So comparing me with them, and telling me that I write too many antibiotic, it’s wrong, your medical advice because it makes me double guess my decision making. And to me, it could harm the patient and benefit the patient.” - PHO Trial, 604
	Older patients will require more or longer antibiotic prescriptions *(Patient population)*	“It seems like the indication for treating cellulitis for five days does seem a little on the short side for me. But I might be seeing maybe more complicated cases of cellulitis because the cellulitis I’m thinking about typically are going to be elderly patients with a lot of concurrent morbidities and they’re getting cellulitis in their legs. Often, these patients are going to be transferred to IV antibiotics after a failure or oral antibiotics. So these are not sort of young, healthy people with cellulitis; these are more advanced, sicker people, and I think the duration of treatment might be longer for them”- PHO Tial, 2296: “One of the big things we have in our practice is a lot of elderly women with chronic UTI issues. So the number of prescriptions I write are probably exceeding the provincial average because I have lots of UTIs every week. I have at least three or four, so it just – of course there’s no alternative treatment for urinary tract infections.” – OH Trial, 28, high prescriber “The expectation of using antibiotics only according to guidelines, is a bit theoretical … but it’s not always possible in real life situations when there is uncertainty and when there’s risks that come with not prescribing for the patients. And sometimes it’s fine. And sometimes it’s fine to take the risk, because you have a backup plan, or you can educate the patient what to do if it develops. But sometimes you can’t because the patient is too frail, or there’s too many complexities and you don’t want to take chances. So you treat them with everything to keep them from decompensating or ending up in the Emergency.” - PHO Trial, 4299
	Easier not to prescribe with patients where there is trust and a relationship *(practice characteristics)*	“If I’ve known the patients long enough that I can communicate that I don’t think they need an antibiotic, so most of them, they’re still with me and trust me.” -PHO 1797: “I think at this point in time, I’m, I’ve had more tools at my disposal and longer relationships with these people where I can encourage them to, and they can trust me that when I say if you just need to come back in three days, I’ll be available and you can get in to see me in three days. And I think that has helped. So I think I’m more able to withstand that pressure.” -PHO, 4255: “When it’s in my own practice with patients I know and I have a longitudinal relationship, then I think there’s a lot more trust and better communication. At least better communication received from the patient because they know me so there’s – I think I’m more successful at the communication piece of why an antibiotic is not required.” OH Trial, 163, high prescriber

### Limited engagement with the antibiotic feedback leads to an information-intention gap

Many physicians did not feel the information in the reports warranted attention, leading to limited interaction with the A&F. Such physicians often explained that aggregated data on antibiotic prescribing rates did not reflect the complexity of their clinical encounters. Some emphasized that it is more important to address specific clinical tasks: “*Guidelines are guidelines*,* patients are patients*” (PHO Trial, 1797). Many also justified limited interaction with the A&F based on beliefs about the controllability of antibiotic resistance:

“Because [resistance] that’s a systemic, holistic problem that needs to be addressed at a systemic level. It is not something that can be approached on a case-by-case basis. It’s just too complex.” – PHO Trial, 1088, unadjusted, no harms.

Perception and acceptance of A&F on antibiotics were closely intertwined. Some physicians who did interact with the A&F described uncertainty around the accuracy of the data, which influenced their perception of the data. They noted the potential contribution of dispensed ‘wait-and-see’ prescriptions to their data (i.e., a practice of prescribing with instructions only to use if things get worse) and concern regarding the data only representing patients over 65 skewing the representation of their clinical performance.

### Intention-behaviour gap exacerbated by clinical uncertainty and competing priorities

Strong beliefs in self-efficacy influenced the *intention* to change prescribing (regardless of the data in the A&F). Most physicians expressed their intention to practice following guidelines and that they were already doing their best to minimize inappropriate prescribing. Many felt that improvement would be infeasible, and this perceived (in)capability interrupted a search for new opportunities.

“I felt I was judicious about my antibiotics, and I always try to persuade people out of it. So I don’t know if it’s going to make a difference on how I interact with patients because I felt I was already doing my best.” – OH Trial, 28, high prescriber.

Many factors affecting prescribing were seen as not amenable to change, including the difficulty of prescribing appropriately when there was limited time with a patient and/or when there was no established trusting relationship with a patient. This was especially notable in settings like the emergency departments and walk in clinic compared to comprehensive family practice,

“The time constraints have an affect. Especially if the patient is insistent. I don’t have time, to argue. I was talking to a colleague, and they said – sometimes I don’t have time to sit down for 20 minutes to educate them why antibiotics is not good. So you just give it to them.”- OH Trial 26, low prescriber.

“When it’s in my own practice with patients I know, and I have a longitudinal relationship, then I think there’s a lot more trust and better communication…But when it’s a one-off episodic encounter, in urgent care: A) I may not know the patient’s medical history; B) the patients sometimes are sicker; C) they don’t know me, and they may not trust my advice with saying no, when they may have previously received antibiotics from other physicians in the exact scenario.” -OH trial 163, high prescriber.

## Discussion

Our theory-informed qualitative process evaluation examined why and how family physicians used antibiotic A&F interventions. Our results explored the impacts of intervention components. We identified unique factors creating information-intention gaps (when A&F fails to encourage recipients that change is necessary), and intention-behaviour gaps (when intentions formulated in response to A&F are not rendered into action). Although the trial results showed that any A&F exposure led to a reduction in antibiotic prescribing by 5%, our qualitative analysis helped explain the lack of differences seen across intervention arms.

We found that the main driver of the information-intention gap was a common belief that the aggregated data did not account for the unique clinical context, including geographic location (e.g., rural), practice setting (e.g., emergency room or walk-in clinic), or patient characteristics. Our findings suggest that providing physicians with ‘adjusted’ comparator data did not address concerns regarding the relevance of the data to their clinical context. Some physician participants did not notice the adjusted comparator statement in the A&F. For others, it is possible that a statistical adjustment was not what physicians needed for acceptance. Instead, it was about the face validity of results and the reassurance that their prescribing data was being directly compared to physicians with similar practice, whether it was geographic, clinical mix or practice setting. These results can inform future antibiotic A&F programs as they highlight a crucial need to address the common refrain that, “my practice is different” within an A&F strategy. Although trial effects did not vary by these sub-groups, we found that family physicians who worked in rural settings, emergency rooms, or walk-in clinics seemed less accepting of feedback. These findings indicate that it may be worthwhile to tailor A&F interventions to these groups so that their contexts can be addressed, and their comparison data is deemed relevant. The main barriers of the intention-behaviour gap were beliefs that it is challenging to prescribe appropriately in situations with time constraints, compounded by not having a prior relationship with the patient. Many family physicians believed they were already doing everything possible to limit antibiotic initiation. Our findings are similar to those that found physicians questioned the A&F data’s reliability and validity [14, 15] and physicians in another study complained of cognitive overload [14]. Another Canadian study of multi-faceted antimicrobial stewardship in primary care in Toronto, reported a similar response to our research, with physicians describing that they used antibiotics judiciously already, and barriers to changing behaviours included inadequate time during clinical encounters [16].

It is important to consider how our findings intersect with the CP-FIT three core prepositions: (1) health care professionals and organisations have a limited capacity to engage with feedback, (2) these parties have strong beliefs about how patient care should be provided that influence their interactions with feedback, and (3) feedback that directly supports clinical behaviours is most effective. Our results explain that likelihood of physicians to engage are impacted by the perceived importance of antibiotic resistance, perceived controllability, and with perceptions of the data accuracy. In the context of Ontario primary care, engaging with quality data is seen as an ‘extra’ activity, above and beyond the ‘core’ clinical work of conducting patient encounters, and are not incentivized in the health system or micro system where they work. In such a context, careful attention to make engagement easy and to limit any negative perceptions about the data becomes critical. Further, it is crucial that data are presented in a fashion that align with existing strong beliefs about how patient care should be provided, such as stratification by disease type or setting. Interestingly, our fidelity survey, reported elsewhere, indicate low engagement with the letter and out of 135 randomly sampled physicians in the intervention group, 41 (30%) either did not receive or were unsure if they received the intervention [17].

Our qualitative data support the quantitative data from the trial showing that the letter made a significant impact on duration of antibiotics. The duration data involved education – as it presented new information for some physicians. In contrast, the initiation data was not about addressing a knowledge gap; the physicians seemed to know the guideline recommendations, and the feedback needed to convince them that there may be room for prescribing fewer antibiotics. The effort to design the feedback components to encourage recipients to accept this information seems to have not been successful; likewise, co-interventions such as the viral prescription pad or harms information that were tested to help put an intention into action did not achieve this effect. Unlike antibiotic initiation, which also showed reductions in the trial, making changes to antibiotic duration was deemed readily feasible. Identifying the most amenable targeted behaviours to A&F remains a topic for ongoing research.

Other qualitative research has described that recipients of A&F were not prompted to improve because their performance was ‘in the middle’ [18–20]. Some research found it was effective to compare to a “top performing group” [21]; however, other research reported similar issues that clinicians considered high benchmarks unachievable and questioned or disengaged from the feedback [18, 22]. Nevertheless, it is common for A&F to compare clinicians to the average of their peer group [23]. Our findings suggest the need to evaluate tailored feedback reports where performance targets are customized to the recipient.

We found that physicians described the viral prescription pad being ‘useful’, however they also reported not ‘using’ it. We can infer that the physicians appreciated the viral prescription pad as a reminder of ways in which they might structure their conversations regarding viral illness with their patients, but not that they intended to incorporate the actual artifact into their encounter. Considering how commonly viral prescription pads are recommended [24], further work should explore why physicians did not use the pad but rather perceived it a reminder.

Our study has several strengths. The current study was embedded within a pragmatic randomized trial, allowing us to explore the quantitative data obtained in real-world settings qualitatively. This created a synergistic mixed-methods approach in which quantitative and qualitative complemented and strengthened each other. This study has some significant limitations to note. Interviews were only conducted with physicians who agreed to participate; therefore, they cannot reflect all family physicians who received the intervention. Furthermore, our team evaluating the feedback intervention was also involved in implementing the intervention, which may have introduced bias to data collection and analysis. Finally, the impact of the COVID-19 pandemic must also be highlighted, as it led to altered work practices and increased stress among participants.

## Conclusion

Our results indicate that many prescribers justified their disengagement from A&F because they perceived their practice was unique. Those who accepted the feedback often described a perceived inability to improve. Our findings suggest that future research should develop and test tailored reports that acknowledge the prescriber’s context and provide personalized performance targets and recommendations, specifically in collaboration with those most likely to disengage.

## Supplementary Information

Supplementary Material 1.

Supplementary Material 2.

## Data Availability

Data sharing not applicable to this article as no datasets have been generated yet.
