# Supplementary material for: Process evaluation of two large randomized controlled trials to understand factors influencing family physicians’ use of antibiotic audit and feedback reports
Source: Implement Sci. 2024 Sep 16;19:65. doi: 10.1186/s13012-024-01393-5 (PMC11403851; doi:10.1186/s13012-024-01393-5)
Supplement: Supplementary file 2 — Supplementary Material 2. [file 13012_2024_1393_MOESM2_ESM.docx]

**Appendix 2: The CP-FIT feedback cycle components and associated interview questions**

| **CP-FIT feedback cycle component** | **Definition** | **Interview question** |
| --- | --- | --- |
| Goal setting | CP-FIT hypothesises that feedback is more effective when the clinical performance standards are considered important and relevant to recipients’ roles. | Did seeing this data cause you to set any goals around your antibiotic prescribing or the way you communicate with patients?  Did seeing this information (duration, harms, viral rx pad, communication tips, virtual care) cause you to set any goals around your antibiotic prescribing or the way you communicate with patients? |
| Data collection and analysis | Automated data collection and analysis processes are generally recommended. Manual collection and analysis are often hindered by a lack of time or skills. | What did you think about the data and where it came from?  Did you check the data and where it came from?  Do you think its accurate?  What did you think about this information (duration, harms) and where it came from? Do you think its accurate? |
| Feedback | Current best evidence supports more frequent provision of feedback. Data should also be as recent as possible, which may enhance subsequent cycle components (Acceptance, Intention, and behaviour) and encourage identification of suboptimal performance. Other relevant factors include problem-solving and action planning, i.e. helping recipients identify and introduce solutions to improve. | N/A |
| Interaction | This component includes the method of delivery and how recipients interact with the feedback, e.g. is it delivered directly to clinicians or do they need to seek it out? | What did you think of your personal antibiotic prescribing data?  Did you look at it? What did you learn from the data in the letter?  Was it easy to understand?  Did you read the section on antibiotic duration/harms/communication tips/viral prescription pad/virtual care? Was it easy to understand?  What did you think of the letter overall?  Regarding the time it took to read the letter? Are you glad that you received it? Was it helpful? |
| Perception | Feedback is more effective when it is user-friendly. Provision of a comparator (e.g. showing performance benchmarked against appropriate others) is considered to facilitate the perception, Intention, and behaviour components. | In the letter, your results are displayed in comparison to other family physicians - can you walk me through what you thought of this comparison?  In your opinion, what would you like to see as a comparator?  What did you think of the information on the achievable target?  What did you think of information given in the letter that discussed duration/harms/communication tips/viral prescription pad/virtual care?  What did you think about the communication suggestions? Prompt: Feasible? Realistic? Relevant to your Practice?  Is the viral prescription pad a useful tool? |
| Verification | A potential component between perception and Acceptance where, if the feedback permits, recipients can explore the data underlying performance. | What did you think about your prescribing data and where it came from?  Did you check the data and where it came from? Do you think its accurate? |
| Acceptance | Acceptance is facilitated when recipients believe the feedback presents a true representation of their performance. Users are more likely to engage with credible feedback, which facilitates several cycle components. | What did you think about your prescribing data and where it came from?  Did you check the data and where it came from?  Do you think its accurate?  In the letter, your results are displayed in comparison to other family physicians - can you walk me through what you thought of this comparison? In your opinion, what would you like to see as a comparator?  What did you think of the information on the achievable target?  What did you think of information given in the letter that discussed duration/harms/communication tips/viral prescription pad/virtual care?  What do you think about antimicrobial resistance? Is it something you consider when you are prescribing antibiotics? Do you discuss it with your patients? |
| Intention | Ideally, recipients form Intentions to take actions to improve performance in response to the feedback. | After seeing your prescribing data, did you consider making any changes regarding your patient interactions when you are considering an antibiotic? Did you consider making any changes to the way you prescribe?  Do you intend to make any changes or have you made any plans to change the way you practice after seeing this information on harms, duration, communication tips, viral prescription pad, or virtual care?  Do you intend to use the viral prescription pad? Why or why not? |
| Behaviour | Feedback that has been received, understood, and accepted will ideally be followed by a planned behavioural response. A distinction is made between patient-level responses, i.e. relating to the care of individuals, and those at the organisational-level with impacts across the wider healthcare system. | If you did make plans to change your prescribing – did those plans pan out?  Have you used the information provided when either making decisions regarding antibiotic prescribing or communication with patients?  After seeing the information, did you end up changing the way you practice?  Have you used the viral prescription pad?  Did you download the viral prescription pad into the EMR? Why or why not?  Is antibiotic prescribing behavior something you monitor? |
| Clinical performance improvement | Organisation-level behaviours are associated with greater clinical performance improvement potential as they enable multiple patient-led behaviours by enhancing the clinical environment in which they occur. | Have you discussed the letter with anyone? Prompt: pharmacist; other physicians  If YES:  Can you talk me through how that conversation went? Prompt: Who was it with? Why that person? Was it useful?  If NO (TO ANYONE OR GROUP):  Why not? Prompt: Barriers  What would encourage you to discuss your letter with others? Prompt: an individual or a group? |
| Unintended consequences | CP-FIT acknowledges the potential for both positive and negative unintended outcomes of feedback interventions. Examples include improved record-keeping, or manipulation of patient populations to artificially improve performance, respectively. | N/A |
